# Recovery of Staphylococci from Teatcups in Milking Parlours in Goat Herds in Greece: Prevalence, Identification, Biofilm Formation, Patterns of Antibiotic Susceptibility, Predictors for Isolation

**DOI:** 10.3390/antibiotics12091428

**Published:** 2023-09-10

**Authors:** Charalambia K. Michael, Daphne T. Lianou, Katerina Tsilipounidaki, Dimitris A. Gougoulis, Themistoklis Giannoulis, Natalia G. C. Vasileiou, Vasia S. Mavrogianni, Efthymia Petinaki, George C. Fthenakis

**Affiliations:** 1 Veterinary Faculty, University of Thessaly, 43100 Karditsa, Greecedlianou@vet.uth.gr (D.T.L.);; 2 Department of Microbiology, University Hospital of Larissa, 41110 Larissa, Greece; 3 Faculty of Animal Science, University of Thessaly, 41110 Larissa, Greece

**Keywords:** antibiotic resistance, biofilm, mastitis, methicillin, milking parlour, goat, sheep, *staphylococcus*, teatcup

## Abstract

The objectives of this work are (a) to describe staphylococci on the teatcups of milking parlours in goat farms and identify predictors for the presence of staphylococcal isolates on the teatcups, (b) to evaluate relationships with total bacterial counts and somatic cell counts in bulk-tank milk, and (c) to establish patterns of susceptibility to antibiotics for the staphylococcal isolates and identify predictors for the recovery of resistant isolates. In a cross-sectional study of 66 goat farms across Greece, swab samples were collected from 303 teatcups (upper and lower part) for staphylococcal recovery, identification, and assessment of biofilm formation. Details regarding health management on the farms (including conditions in the milking parlour) and the socio-demographic characteristics of farmers were collected by means of a structured questionnaire. A total of 87 contaminated teatcups (28.7%) were found on 35 goat farms (53.0%). Staphylococci were more frequently recovered from the upper than the lower part of teatcups: 73 versus 43 teatcups, respectively. After identification, 67 staphylococcal isolates (i.e., excluding similar isolates) were recovered from the teatcups; *Staphylococcus aureus*, *Staphylococcus capitis*, and *Staphylococcus equorum* predominated. Of these isolates, 82.1% were biofilm-forming. In multivariable analysis, the annual incidence of clinical mastitis in the herd emerged as the only significant factor associated with the isolation of staphylococci from the teatcups. Of the 67 isolates, 23 (34.3%) were resistant to at least one antibiotic, and 14 (22.4%) were multi-resistant. Resistance was found most commonly against penicillin and ampicillin (22.4% of isolates), fosfomycin (17.9%), clindamycin (14.9%), erythromycin, and tetracycline (13.4%). In multivariable analysis, the annual incidence of clinical mastitis in the herd and the use of detergent for parlour cleaning emerged as significant factors associated with the isolation of staphylococci resistant to antibiotics.

## 1. Introduction

### 1.1. Significance of the Milking Parlour in the Development of Mastitis

The most important equipment in a dairy farm environment is the milking parlour. Teatcups in a milking parlour can contribute to the dissemination of bacteria into the mammary gland [1,2]. Indeed, Liagka et al. [3] have recently indicated that bacteria on teatcups can enter into the teat of ewes during the milking routine. Hence, there is interest in the study of staphylococcal populations on teatcups of milking parlours, as staphylococci are the primary pathogens causing mastitis in small ruminants [4].

All these can be exacerbated during a malfunction or improper use of the milking machine, as it may affect teat status, causing changes or even lesions, which would facilitate the onset of mammary infection [1,2].

### 1.2. Importance of Biofilm-Formation in Staphylococci Associated with Mastitis

Staphylococci are the primary causes of mastitis in dairy small ruminants and are responsible for over 65% of cases of infection [4]. Various factors can contribute to the virulence of these bacteria and can participate in the pathogenetic process of mastitis.

Among the virulence determinants, biofilm formation improves the survival of staphylococcal isolates, dissemination in the milking parlour, and adherence to epithelial cells therein. Formation of biofilm by staphylococci leads to the expansion of the bacteria, offers reduced susceptibility to antimicrobial agents, and promotes bacterial survival from mammary defences [5,6,7]. The main constituents of the biofilm matrix are polysaccharides and peptides [8].

### 1.3. Antibiotic Resistance of Staphylococci Recovered from Goat Milk in Greece

In a previous paper [9], we reported the patterns of staphylococci (frequency of isolation, species identified, antibiotic resistance) recovered from bulk-tank milk in goat herds in an extensive, countrywide investigation in Greece, as well as their antibiotic susceptibilities. The findings of that study indicated that most staphylococcal species recovered from bulk-tank milk samples were identified as species often associated with mammary infection [10]. Staphylococcal species of possibly environmental origin were also recovered, suggesting that they might have originated from other sources, e.g., farm equipment [9].

In that study, it was reported that staphylococci isolated from the bulk-tank milk of goat herds in Greece were most often resistant to penicillin, ampicillin, and fosfomycin. It was postulated that the recovery of resistant staphylococci in milk intended for human consumption raises concerns, as, potentially, the genetic material of these resistant staphylococci, which is not destroyed during the thermal processing of milk, might possibly be transferred to humans [11,12]. These genes could be incorporated into other bacteria, which constitute a part of the normal flora of humans, leading to the dissemination of resistance genes.

### 1.4. Objectives of the Study

The objectives of this work are (a) to describe staphylococci on the teatcups of milking parlours in goat farms and identify predictors for the presence of staphylococcal isolates on the teatcups, (b) to evaluate relationships with total bacterial counts and somatic cell counts in bulk-tank milk, and (c) to establish patterns of susceptibility to antibiotics for the staphylococcal isolates and identify predictors for the recovery of resistant isolates.

## 2. Results

### 2.1. Recovery of Staphylococcal Isolates from Teatcups

Staphylococci were recovered from the teatcups of milking parlours of 35 goat herds (53.0%; 95% CI: 41.2%–64.6%).

Staphylococci were recovered from 87 of the 303 teatcups sampled (28.7%; 95% CI: 23.9%–34.1%). They were isolated significantly more frequently from the upper than the lower part of the swabbed teatcups: 70 (23.1%; 95% CI: 18.7%–28.2%) versus 42 (13.9%; 95% CI: 10.4%–18.2%) teatcups, respectively (*p* = 0.003).

Overall, 116 staphylococcal isolates were recovered. Of these, 73 (62.9%) were recovered from the upper and 43 (37.1%) from the lower part of the teatcups sampled; the mean number of staphylococcal isolates recovered from each part of the teatcups did not differ between the two parts of each teatcup: 1.04 versus 1.02 per contaminated part of teatcup, respectively (*p* = 0.60).

### 2.2. Identification of Staphylococcal Isolates from Teatcups

After full identification, a total of 67 unique isolates (i.e., excluding similar isolates), which belonged to 17 staphylococcal species, were recovered from the teatcups. Among these, 51 isolates (belonging to 16 species) were recovered from the upper part, and 32 isolates (belonging to 13 species) were recovered from the lower part of teatcups (16 isolates were recovered from the upper part and the lower part of the same teatcup).

Of the 67 isolates, 6 (9.0%) were *Staphylococcus aureus*, and the other 61 (91.0%) were non-aureus isolates. Among these, the most frequently identified species were *S. capitis* (*n* = 11, 16.4%) and *S. equorum* (*n* = 10, 14.9%) (Table 1). During bacterial identification, results showed that all isolates had a score > 2, which indicates staphylococcal identification at the species level with high accuracy. For the five staphylococcal species most frequently identified (among non-aureus isolates), there were no statistically significant differences in the frequencies of isolation from the upper or the lower part of teatcups (*p* > 0.14 for all comparisons).

### 2.3. Biofilm Formation by Staphylococci Recovered from Teatcups

Biofilm-forming staphylococci were recovered from 82 teatcups (94.3% of contaminated teatcups; 95% CI: 87.2%–97.5%) from 33 herds (94.3% of those from which staphylococci were isolated; 95% CI: 81.4%–98.4%). There was no statistically significant difference between the upper and lower part of contaminated teatcups, in the proportion of contaminated teatcups among which biofilm-forming isolates were recovered: 88.6% (62/70) versus 90.5% (38/42), respectively (*p* = 0.75).

Of the 67 unique isolates recovered from the teatcups (as detailed in Section 2.2), 55 (82.1%; 95% CI: 71.3%–89.5%) were biofilm-forming. There was no statistically significant difference in the proportion of biofilm-forming isolates obtained from the upper or the lower part of teatcups: 43/51 (84.3%) versus 29/32 (90.6%), respectively (*p* = 0.41). Also, there was no statistically significant difference in the proportion of biofilm-forming isolates between the various staphylococcal species (*p* = 0.42) (Table 2).

### 2.4. Variables Associated with Recovery of Staphylococcal Isolates from Teatcups

The detailed results of the univariable analysis for the isolation of staphylococci from teatcups are in Table S1. Among the variables included in the multivariable analysis (Table S1), only the annual incidence of clinical mastitis in the herd emerged as a significant factor (*p* = 0.043) (Table 3).

### 2.5. Associations with Total Bacterial Counts and Somatic Cell Counts in Bulk-Tank Milk

The median (interquartile range) for total bacterial counts and somatic cell counts in the bulk-tank milk of the farms in the study were 677 × 10^3^ (1170 × 10^3^) cfu mL^−1^ and 0.899 × 10^6^ (0.648 × 10^6^) cells mL^−1^, respectively.

There was some association between the isolation of staphylococci from teatcups and total bacterial counts/somatic cell counts in the bulk-tank milk. Specifically, in herds with total bacterial counts over 2000 × 10^3^ cfu mL^−1^ (*n* = 11) or with somatic cell counts over 1.250 × 10^6^ cells mL^−1^ (*n* = 17), there was a significantly higher proportion of contaminated teatcups than in herds with total bacterial counts or somatic cell counts below these values (*p* = 0.002 and 0.043, respectively) (Table 4).

### 2.6. Isolation of Antibiotic-Resistant Staphylococci

Resistant (to at least one (any) antibiotic) or multi-resistant staphylococci were isolated from 14 (21.2%, 95% CI: 13.1%–32.5%) or 11 (16.7%, 95% CI: 9.6%–27.4%) herds, respectively.

Of the 67 staphylococcal isolates recovered from the teatcups (as detailed in Section 2.2), 23 (34.3%, 95% CI: 24.1%–46.3%) (1 *S. aureus* and 22 non-aureus isolates) were resistant to at least one (any) antibiotic (*p* = 0.34 for comparison between *S. aureus* and non-aureus staphylococci; *p* = 0.53 for comparison between the various non-aureus species).

Further, 14 isolates among the 23 that were found to be resistant to at least one antibiotic (22.4%, 95% CI: 14.1%–33.7%, of all isolates recovered from the teatcups; 60.9%, 95% CI: 40.8%–77.8%, of isolates resistant to at least one antibiotic) were multi-resistant (all non-aureus isolates, *p* = 0.19 for comparison between *S. aureus* and non-aureus staphylococci; *p* = 0.35 for comparison between the various non-aureus species). Details are in Table 5.

At the isolate level, resistance was found most commonly against penicillin and ampicillin (15 isolates, 22.4% of all isolates), fosfomycin (12 isolates, 17.9% of all isolates), clindamycin (10 isolates, 14.9% of all isolates), erythromycin, and tetracycline (9 isolates for each antibiotic, 13.4% of all isolates). Resistance to oxacillin was detected in 4 isolates (6.0%) (Table S2). There was no statistically significant difference in the proportion of resistant staphylococci isolated between the upper and lower parts of the teatcups: 18/51 (35.3%) versus 9/32 (28.1%) (*p* = 0.50).

At the herd level, staphylococci resistant to penicillin and ampicillin were isolated from 12 (18.2%, 95% CI: 10.7%–29.1%) milking parlours, staphylococci resistant to erythromycin, fosfomycin, and tetracycline from 9 (13.6%, 95% CI: 7.4%–23.9%) parlours and staphylococci resistant to clindamycin from 8 (12.1%, 95% CI: 6.3%–22.1%) parlours. Staphylococci resistant to oxacillin were isolated from 4 (6.1%, 95% CI: 2.4%–14.6%) milking parlours.

Among staphylococcal species of which at least two isolates were recovered, *S. equorum* was found to be resistant most commonly to ampicillin, clindamycin, erythromycin, penicillin, and fosfomycin (4/10, 4/10, 4/10, 4/10, and 2/10 isolates, respectively), *S. saprophyticus* was found to be resistant most commonly to fosfomycin, ampicillin, fusidic acid, and penicillin (3/6, 2/6, 2/6, and 2/6 isolates, respectively), *S. lentus* was found to be resistant most commonly to ampicillin, ciprofloxacin, clindamycin, oxacillin, and penicillin (2/6 isolates for each antibiotic), and *S. capitis* to fosfomycin (3/11 isolates) (Table S2).

Finally, there was no statistically significant difference in the proportion of biofilm-forming isolates among resistant (17/23, 73.9%) and susceptible (38/44, 86.4%) isolates (*p* = 0.21).

### 2.7. Variables Associated with Recovery of Resistant or Multi-Resistant Staphylococcal Isolates from Teatcups

#### 2.7.1. Isolation of Oxacillin-Resistant Staphylococcal Isolates

The detailed results of the univariable analysis for the isolation of oxacillin-resistant staphylococci from teatcups are in Table S3. In the multivariable analysis, no variable emerged with a significance (*p* > 0.09).

#### 2.7.2. Isolation of Staphylococcal Isolates Resistant to at Least One Antibiotic

**Figure 1:**
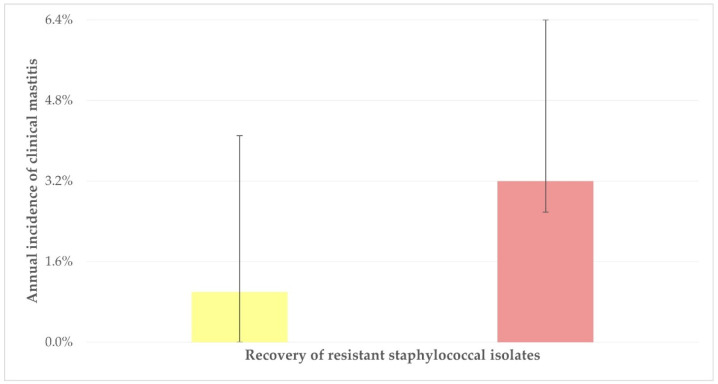
Median incidence of clinical mastitis among herds from which resistant staphylococcal isolates were (red bar) or were not (yellow bar) recovered.

The detailed results of the univariable analysis for the isolation from teatcups of staphylococci resistant to at least one antibiotic are in Table S4. In the multivariable analysis, the following variables emerged with significance: (a) annual incidence of clinical mastitis in the herd (*p* = 0.017) and (b) omission of the use of detergent for parlour cleaning after the milking sessions (*p* = 0.034) (Table 6). The median incidence of clinical mastitis was 3.2% (interquartile range: 3.8%) and 1.0% (4.1%) among herds from which resistant staphylococcal isolates were or were not recovered (*p* = 0.04) (Figure 1). It is also noted that there was a tendency for significance for the application of post-milking teat disinfection (*p* = 0.055).

#### 2.7.3. Isolation of Multi-Resistant Staphylococcal Isolates

The detailed results of the univariable analysis for the isolation of oxacillin-resistant staphylococci from teatcups are in Table S5. In the multivariable analysis, the omission of the use of detergent for parlour cleaning after the milking sessions emerged as a significant variable (*p* = 0.034) (Table 7). It is also noted that there was a tendency for significance for the application of post-milking teat disinfection (*p* = 0.07).

## 3. Discussion

### 3.1. Isolation of Staphylococci from Teatcups

During the study, samples were collected at the end of the milking routine, subsequent to the completion of the post-milking cleaning of the parlour. That way, we aimed to recover only isolates that might have remained on the teatcups. The findings of the study indicate that, despite post-milking cleaning of the parlour, staphylococci are present on the surface of the teatcups. Most of these isolates were biofilm-forming, which can explain their increased presence on the teatcup surface. Given the above, one may reasonably postulate that during the actual milking procedure (i.e., when animals are going in and out of the milking parlour) the frequency of infection of the teatcups would be much higher.

The more frequent recovery of staphylococcal isolates from the upper part of the teatcups is likely the result of the increased exposure of that part of the teatcup to external contamination. In contrast, staphylococci isolated from the lower part of teatcups likely have originated more often from animals. This is the result of milk flowing out of the teat during milking and coming in contact with the lower part of the teatcups.

The accuracy of using Matrix-Assisted Laser Desorption/Ionization Time-of-Flight Mass Spectrometry for the identification of the various staphylococcal species has previously been confirmed in various studies, e.g., Dubois et al. [13] and Mahmmod et al. [14]. The present findings of a high score during the identification of the isolates confirm the accuracy of the identification procedure and can be allied to the previous findings above to confirm correct staphylococcal identifications at the species level.

It is noted that many of the staphylococcal species identified are confirmed frequent mammary pathogens, e.g., *S. aureus*, *S. caprae*, *S. chromogenes*, *S. epidermidis*, *S. equorum*, *S. lentus*, *S. simulans*, *S. warneri*, *S. xylosus* [15]. This lends support to the results of the multivariable analysis, in which the annual incidence of clinical mastitis in the herd emerged as a significant factor for the recovery of staphylococci from the teatcups: a high incidence of the infection in the herd leads to increased shedding of staphylococci in milk, and thus, after milking, the bacteria would be recovered more often from the teatcups, as found in the present study. Nevertheless, the recovery of other staphylococcal species, less frequent causal agents of mastitis, and predominantly human pathogens (e.g., *S. saprophyticus* [16]) indicates the possibility of cross-transmission between people working on the farm and the animal environment.

Many factors were evaluated for potential association with staphylococcal isolation. These included variables related to the general farm management and variables related to the procedures applied at the milking parlour, for example, the pulsation rate or the frequency of teatcup change. However, none of these was found to be important; only the increased incidence of mastitis emerged to have a significant association with the presence of staphylococci in the teatcups.

In cases of increased incidence of clinical mastitis on a farm, the incidence of subclinical mastitis would also be high [4]. In such cases, a high number of animals on the farm would be excreting staphylococci in the milk. These bacteria can infect other animals that go into the parlour, disseminating the infection within the herd. It is noteworthy that in a recent study, Liagka et al. [3] reported that during the milking procedure after milking animals with mastitis, there was a 10-times higher odds ratio for transmission of staphylococci to the animal milked subsequently to the infected one, through the dissemination of the bacteria on the teatcups.

Biofilm formation in a bacterial community leads to the attachment of the bacterial cells onto a surface or an interface or between one another. The ability for biofilm formation by staphylococci helps them to colonize onto inert surfaces and improves their ability to adhere to host cells. Biofilm formation is linked to the formation of extracellular polysaccharides, which play an important role in bacterial adhesion. The presence of biofilm-forming staphylococci can be associated with two further issues. First, biofilm-forming staphylococci can attach easily to the skin of the teat of animals at the time of milking, and subsequently, they may invade the teat duct of these animals and cause mastitis [3,17]. Indeed, biofilm-forming staphylococcal isolates have been found to amount for as much as 40%–43% [18] to 91%–98% [19] of all mastitis-causing staphylococcal isolates in small ruminants. However, it has been postulated that biofilm formation by staphylococci plays a role only in the infection process and the colonization of the mammary gland and is not involved in tissue damage and the development of inflammatory reactions during mammary infections [20,21]. On the other hand, the presence of biofilm-forming bacteria on dairy equipment is significant because these bacteria multiply and expand quickly on teatcups (over-tripling the surface covered within 12 h) [22] and can contribute to contamination of the milk produced on a farm that is destined for human consumption. Indeed, the formation of biofilm by bacteria during the stage of milk production or storage may lead to potential problems, given that biofilm-forming bacteria may survive during some heat-processing steps (e.g., high-temperature pasteurisation) [23,24].

### 3.2. Antibiotic Resistance of Staphylococcal Isolates

Overall, the antibiotic resistance patterns are, in general, similar to patterns of resistance found in staphylococcal isolates obtained in Greece from clinical samples (i.e., from cases of mastitis), which indicated more frequent recovery of isolates resistant to penicillin, tetracycline, and ampicillin [25]. All the findings are in line with the results of a study into the usage of antibiotics against mastitis on small ruminant farms in Greece, which reported these drugs as the ones most frequently administered [26]. However, fosfomycin is not licenced for veterinary use in Greece and thus is not prescribed for the treatment of animal infections but only against infections in people. Hence, the detection of isolates resistant to fosfomycin is puzzling, and one may possibly explain this by postulating a possible human origin of such isolates (e.g., from people working on the farm, as mentioned above).

In farms with increased (>1% annually) incidence of clinical mastitis, a higher odds ratio was found for the isolation of resistant staphylococci from the teatcups. It can be postulated that this is likely the consequence of increased antibiotic administration in such farms, used to treat the high number of mastitis cases, as antibiotic resistance is accelerated by the overuse of antibiotics when bacteria evolve to evade the effect of antibiotics through multiple mechanisms [27,28].

The omission of using detergents during parlour cleaning was found to be uncommon among farms included in the study. This was also found to be associated with a higher odds ratio for the isolation of resistant staphylococci. One may postulate that the association between detergent use and antibiotic resistance is driven by the effect of detergents on the dynamics and growth of bacterial populations. In general, the use of detergents leads to a reduction in bacterial populations, even antibiotic-resistant isolates [29,30], given that the growth and spread of antibiotic resistance can be significantly affected by the size of bacterial populations [31,32,33]. The link between bacterial population size and the development of antibiotic resistance is crucial for understanding the evolution and spread of resistant isolates [34,35], as resistance mutations or acquisition of resistance genes are relatively uncommon in small bacterial populations, as well as because the emergence of resistance is more likely due to the large numbers of bacteria in larger populations [32]. In such cases, a wider genetic diversity pool increases the likelihood of spontaneous mutations or the presence of resistant strains already prevailing within the population. Consequently, there is a higher possibility that subpopulations of antibiotic-resistant bacteria would develop and spread. The potential for horizontal transfer of resistance genes is also increased by larger bacterial populations [36,37,38]. Larger populations can offer a favourable environment for the transmission of antibiotic resistance genes [36,37,38].

Poor management at the milking parlour and an incorrect milking routine, e.g., omission of using disinfectants, contribute to increased bacterial burdens in the animal environment and, thus, higher risk of infection and, consequently, development of mastitis [39]. Mastitis leads to the overuse of antibiotics for therapeutic purposes [40] and, thus, the development of antibiotic resistance, as well as increased bacterial shedding at the milking parlour during milking, furthering the risk of mammary infection, creating a vicious circle.

## 4. Materials and Methods

### 4.1. Goat Herds

**Figure 2:**
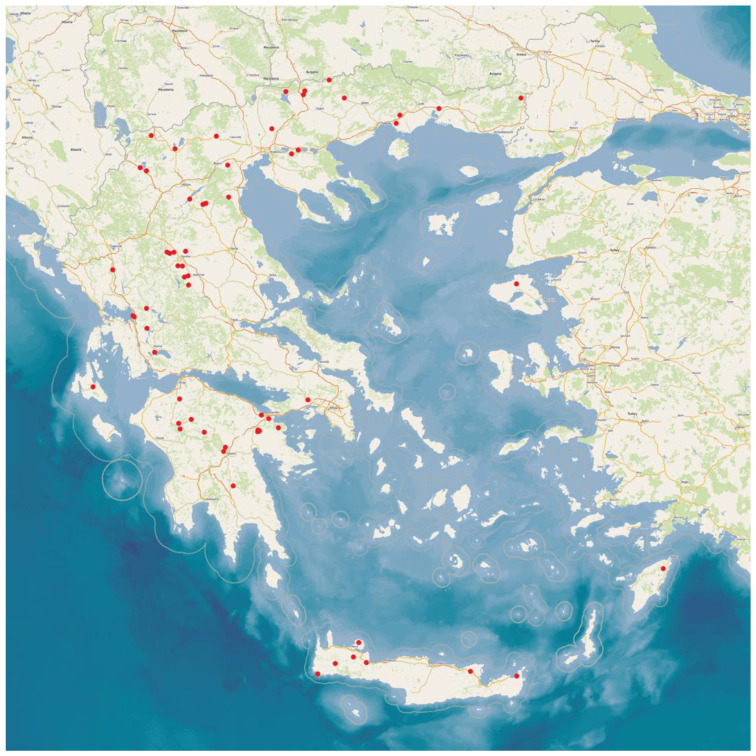
Location of the goat herds around Greece, which were visited for collection of samples and information.

This work is part of a large, countrywide cross-sectional study performed among 119 goat farms throughout Greece, of which 66 had milking parlours. The farms were located in the 13 administrative regions in Greece (Figure 2). All the farms were visited for the collection of samples and information. Veterinarians working with goats across Greece were contacted and asked whether they were interested in collaborating in the investigation; in total, 32 veterinarians participated in the present study. The herds were selected by the collaborating veterinarians on a convenience basis (willingness of farmers to accept a visit by University personnel for an interview and sample collection). The principal investigators (authors C.K.M. and G.C.F.) visited all the study farms for sampling.

An interview with the goatherd was carried out to record husbandry and health management variables in the farm, as well as the characteristics of the milking parlour [41].

### 4.2. Samplings

On each farm, swab samples were taken from the teatcups of the milking parlour. The following sampling protocol was used. In milking parlours with 1 milking unit (*n* = 3), swabs were taken from 2 teatcups; in parlours with 2 to 12 milking units (*n* = 32), swabs were taken from 3 teatcups; in parlours with 2 to 13 to 24 units (*n* = 28), swabs were taken from 6 teatcups; finally, in parlours with 25 to 36 (*n* = 1) or 37 to 48 units (*n* = 2), 9 and 12 teatcups, respectively, were swabbed.

The specific teatcups sampled in each parlour were predetermined using an electronic random number generator. Therefore, in total, 303 teatcups were sampled. Two separate swabs were obtained from each teatcup: one was taken from the upper (approx. 1–1.5 cm deep), and one was taken from the lower (approx. 10–12 cm deep) part of the teatcup. During sampling, the entire circumference of the inner wall of the teatcup was swabbed in a circular manner. Duplicate swab samples were taken, i.e., in total, four sterile swabs were obtained from on each teatcup. The swabs were immersed into transportation medium (Liquid Based Microbiology—LBM; BioMerieux, Marcy-l’-Étoile, France).

All swab samplings were taken after the end of a milking session and the cleaning of the parlour, which was performed following the usual farm routine. Parlour cleaning included washing the parlour with water, cleaning liners, teatcups, clusters, long milk tubes, and pulse tubes, flushing with detergents with acid or alkaline pH, and rinsing.

Finally, bulk-tank milk samples were taken aseptically from each herd for cytological and bacteriological examination. These were collected using sterile plastic single-use pipettes, which were immersed into the tank to withdraw the samples. Four separate samples were collected from the milk tank of each herd using a new pipette for each type and transferred into sterile plastic Universal-type vials for laboratory testing.

Samples were stored at 0.0 to 4.0 °C using ice packs in portable refrigerators. Transportation of samples to the laboratory was made by the investigators and by car; samples collected from herds on the islands were transported as ice-packed accompanying luggage by airplane (Crete, Lesvos, and Rhodes) or by boat (Cephalonia).

### 4.3. Laboratory Examinations

#### 4.3.1. Somatic Cell Counting and Total Bacterial Counting in Bulk-Tank Milk Samples

Two milk samples from each bulk tank were used for somatic cell counting. Two sub-samples were created and processed from each of these two samples so that somatic cell counting (Lactoscan SCC; Milkotronic Ltd., Nova Zagora, Bulgaria) was performed four times, each time in a different sub-sample.

The remaining two milk samples were used for performing total bacterial counting, which was carried out following the procedures detailed by Laird et al. [42]. After completion of sample aliquot withdrawal for microbiological examination, the temperature of the respective samples was measured and was found never to exceed 3.8 °C.

#### 4.3.2. Isolation and Identification of Staphylococcal Isolates

Each of the four swabs obtained from a teatcup (two swabs from the upper and two swabs from the lower part of the teatcup) was cultured in duplicate on 5% sheep blood agar and on *Staphylococcus* selective medium (Mannitol salt agar; BioPrepare Microbiology, Athens, Greece).

All the media were placed for aerobic incubation at 37 °C for 48 h; if there was no growth, they were reincubated for another 24 h. Bacterial isolation and initial identification were performed using standard methods [43,44]. The staphylococcal isolates were finally identified at the species level using Matrix-Assisted Laser Desorption/Ionization Time-of-Flight Mass Spectrometry (VITEK MS; BioMerieux, Marcy-l’-Étoile, France).

#### 4.3.3. Evaluation of Biofilm Formation by Staphylococcal Isolates

The in vitro biofilm formation by the isolates was tested by combining the findings of (a) culture appearance on Congo Red agar plates and (b) results of the microplate adhesion test, as detailed by Vasileiou et al. [21]. In order to assess the culture appearance of staphylococcal isolates, these were cultured on Congo Red agar plates (BioPrepare Microbiology, Athens, Greece), which were incubated aerobically at 37 °C for 24 h [45]. For the assessment of biofilm formation by means of the microplate method, the technique presented by Fabres–Klein et al. [46] was followed based on the principles set by Vasudevan et al. [47]; the method involved the measurement of the absorbance rate after coating microplate wells with bacterial culture incubated with tryptic soy broth [35]. The results of the two methods were combined [21], and the isolates were characterized as biofilm-forming or non-biofilm-forming.

#### 4.3.4. Testing for Susceptibility to Antibiotics

All staphylococcal isolates obtained in the study were assessed for sensitivity to antibiotics. A total of 20 antibiotics were tested: amikacin, ampicillin, ceftaroline, ciprofloxacin, clindamycin, erythromycin, fosfomycin, fusidic acid, gentamicin, linezolid, moxifloxacin, mupirocin, oxacillin, penicillin G, rifampin, streptomycin, teicoplanin, tetracycline, tobramycin, and trimethoprim–sulfamethoxazole.

Testing was carried out by means of the automated system BD Phoenix™ M50 (BD Diagnostic Systems, Sparks, MD, USA).

The interpretation of the results was based on the criteria of the European Committee on Antimicrobial Susceptibility Testing (EUCAST) (http://www.eucast.org).

### 4.4. Data Management and Analysis

#### 4.4.1. Data Management

The detection of at least three staphylococcal colonies on at least one agar plate of those cultured with each swab was considered to indicate the presence of the organism. The two sampling sites of each teatcup (upper and lower part) were evaluated separately. If staphylococci were recovered from at least one of these two sites, the teatcup was described as ‘contaminated’.

Staphylococcal isolates were characterised as biofilm-forming or non-biofilm-forming, according to the combination of the results of the two techniques applied (culture appearance on Congo Red agar and microplate adhesion), as described before [27].

Staphylococcal isolates were characterised as susceptible, susceptible to increased exposure, or resistant to each antibiotic based on the results of susceptibility/resistance testing. Isolates found to be resistant to at least three different classes of antibiotics were classified as multidrug-resistant (multi-resistant) isolates [48].

All similarly identified staphylococcal species from swab samples from teatcups of the same milking parlour had the same biofilm-forming profile and the same antimicrobial susceptibility pattern. Therefore, when the same staphylococcal species was identified from swab samples obtained from the same parlour, it was deemed to be the same organism. Hence, it was taken into account in the relevant calculations only once.

#### 4.4.2. Statistical Analysis

Data were entered into Microsoft Excel and analysed using SPSS v. 21 (IBM Analytics, Armonk, NY, USA). Basic descriptive analysis was performed. Exact binomial confidence intervals (CIs) were obtained.

Comparisons between frequencies were performed by using Pearson’s chi-square test or Fisher’s exact test, as appropriate. Comparisons between continuous data were performed using the Mann–Whitney test or by analysis of variance (one-way or Kruskal–Wallis test), as appropriate.

In total, up to 40 variables related to (a) sampling conditions, (b) general management of the herd, (c) the milking parlour of the herd, and (d) socio-demographic particulars of farmers, were evaluated for the identification of predictors (Table S6). Categories were created for these variables according to the information provided by farmers during the interview.

The outcomes of ‘isolation of staphylococci from teatcups’, ‘isolation of oxacillin-resistant staphylococcal isolates from teatcups of a milking parlour’, ‘isolation of resistant staphylococcal isolates from teatcups of a milking parlour’, and ‘isolation of multi-resistant staphylococcal isolates from teatcups of a milking parlour’ were considered. Exact binomial CIs were obtained. The importance of predictors was assessed by using cross-tabulation with Pearson’s chi-squared test and with simple logistic regression. Then, multivariable models were created. In these, all variables that achieved *p* < 0.20 in the univariable analysis were offered to the model. Variables were removed from the initial model by backward elimination. The *p*-value of removal of a variable was assessed by the likelihood ratio test, and for those with a *p*-value of > 0.20, the variable with the largest probability was removed. This process was repeated until no variable could be removed with a *p*-value of >0.20. The variables required for the various multivariable models are shown in Table S7.

In all analyses, statistical significance was defined at *p* < 0.05.

## 5. Conclusions

The study investigated, for the first time internationally, the isolation of staphylococci from teatcups of milking parlours and their antibiotic resistance profiles, as well as relevant predictors.

Staphylococci were isolated from the teatcups of parlours in goat herds, despite post-milking cleaning of the parlours. Most of the isolates were biofilm-forming, which is a property that helps the bacteria to attach to the teatcups and survive unfavourable environmental conditions.

The increased incidence of clinical mastitis in animals on the farm was associated with the isolation of staphylococci from the teatcups, suggesting that the bacteria originated from infected animals and might further infect healthy ones. It was also associated with the isolation of antibiotic-resistant staphylococci, possibly as the result of increased antibiotic administration as part of treatment for the infection. The omission of detergent use also emerged to be associated with the isolation of multi-resistant staphylococci, possibly because the presence of large bacterial populations on the teatcups may contribute to the horizontal transfer of resistance genes.

## Figures and Tables

**Table 1:** Frequency of recovery of staphylococcal isolates (*n* = 67) from teatcups of milking parlours in goat farms.

Overall		Upper Part of Teatcups		Lower Part of Teatcups	
Staphylococcal Species	*n*	Staphylococcal Species	*n*	Staphylococcal Species	*n*
*S. aureus*	6	*S. aureus*	4	*S. aureus*	5
*S. auricularis*	1	*S. auricularis*	1	*S. capitis*	5
*S. capitis*	11	*S. capitis*	6	*S. cohnii* subsp. *urealyticum*	1
*S. caprae*	1	*S. caprae*	1	*S. epidermidis*	1
*S. cohnii* subsp. *urealyticum*	1	*S. epidermidis*	1	*S. equorum*	3
*S. epidermidis*	1	*S. equorum*	8	*S. haemolyticus*	6
*S. equorum*	10	*S. haemolyticus*	4	*S. kloosii*	1
*S. haemolyticus*	7	*S. kloosii*	1	*S. lentus*	3
*S. kloosii*	1	*S. lentus*	5	*S. pasteuri*	1
*S. lentus*	6	*S. pasteuri*	3	*S. pettenkoferi*	1
*S. pasteuri*	4	*S. pettenkoferi*	2	*S. saprophyticus*	3
*S. pettenkoferi*	2	*S. saprophyticus*	6	*S. sciuri*	1
*S. saprophyticus*	6	*S. sciuri*	2	*S. warneri*	1
*S. sciuri*	3	*S. simulans*	2		
*S. simulans*	2	*S. warneri*	4		
*S. warneri*	4	*S. xylosus*	1		
*S. xylosus*	1				

**Table 2:** Proportion of biofilm-forming staphylococcal isolates recovered from teatcups of milking parlours in 66 goat herds in Greece.

Staphylococcal Species	Proportion of Biofilm-Forming Isolates
*S. aureus*	100% (6/6)
*S. auricularis*	100% (1/1)
*S. capitis*	81.8% (9/11)
*S. caprae*	0.0% (0/1)
*S. cohnii* subsp. *urealyticum*	100% (1/1)
*S. epidermidis*	100% (1/1)
*S. equorum*	80.0% (8/10)
*S. haemolyticus*	71.4% (5/7)
*S. kloosii*	100% (1/1)
*S. lentus*	66.7% (4/6)
*S. pasteuri*	75.0% (3/4)
*S. pettenkoferi*	100% (2/2)
*S. saprophyticus*	100% (6/6)
*S. sciuri*	100% (3/3)
*S. simulans*	100% (2/2)
*S. warneri*	50.0% (2/4)
*S. xylosus*	100% (1/1)
Total	82.1% (55/67)

**Table 3:** Results of multivariable analysis for assessment of the isolation of staphylococci from teatcups of milking parlours in 66 goat herds in Greece.

Variable	Odds Ratio ^1^ (95% Confidence Intervals)	*p*
Annual incidence of clinical mastitis		0.043
≤1% (12/29, 41.4%)	reference	-
>1% (23/37, 62.2%)	2.327 (0.862–6.287)	0.09

^1^ odds ratio calculated against the lowest prevalence associations of variable.

**Table 4:** Proportion of contaminated teatcups in milking parlours, in association with total bacterial counts and somatic cell counts in the bulk-tank milk in 66 goat herds in Greece.

**Total Bacterial Counts in Bulk-Tank Milk**	
≤2000 × 10^3^ cfu mL^−1^ (*n* = 55 herds)	>2000 × 10^3^ cfu mL^−1^ (*n* = 11 herds)
24.8% (61/246 teatcups) ^a^	45.6% (26/57) ^a^
**Somatic Cell Counts in Bulk-tank Milk**	
≤1.250 × 10^6^ cells mL^−1^ (*n* = 49 herds)	>1.250 × 10^6^ cells mL^−1^ (*n* = 17 herds)
25.6% (57/223 teatcups) ^b^	37.5% (30/80 teatcups) ^b^

^a, b^ within the same row: *p* < 0.05.

**Table 5:** Frequency of resistant isolates among different staphylococcal species recovered from teatcups of milking parlours in 66 goat herds in Greece.

Staphylococcal Species	Resistant Isolates	Multi-Resistant Isolates
*S. aureus* (*n* = 6 ^1^)	1 (16.7% ^2^)	0 (0.0% ^2^)
*S. auricularis* (*n* = 1)	1 (100%)	0 (0.0%)
*S. capitis* (*n* = 11)	3 (27.3%)	1 (9.1%)
*S. caprae* (*n* = 1)	1 (100%)	0 (0.0%)
*S. cohnii* subsp. *urealyticum*(*n* = 1)	1 (100%)	0 (0.0%)
*S. epidermidis* (*n* = 1)	0 (0.0%)	0 (0.0%)
*S. equorum* (*n* = 10)	5 (50.0%)	5 (50.0%)
*S. haemolyticus* (*n* = 7)	2 (28.6%)	1 (14.3%)
*S. kloosii* (*n* = 1)	0 (0.0%)	0 (0.0%)
*S. lentus* (*n* = 6)	2 (33.3%)	2 (33.3%)
*S. pasteuri* (*n* = 4)	1 (25.0%)	0 (0.0%)
*S. pettenkoferi* (*n* = 2)	0 (0.0%)	0 (0.0%)
*S. saprophyticus* (*n* = 6)	3 (50.0%)	2 (33.3%)
*S. sciuri* (*n* = 3)	1 (33.3%)	1 (33.3%)
*S. simulans* (*n* = 2)	1 (50.0%)	1 (50.0%)
*S. warneri* (*n* = 4)	0 (0.0%)	0 (0.0%)
*S. xylosus* (*n* = 1)	1 (100%)	1 (100%)

^1^ total no. of isolates recovered and tested; ^2^ proportion of isolates resistant to at least one (any) antibiotic or of multi-resistant isolates among all isolates of that species.

**Table 6:** Results of multivariable analysis for assessment of the isolation of resistant staphylococci from teatcups of milking parlours in 66 goat herds in Greece.

Variables	Odds Ratios ^1^ (95% Confidence Intervals)	*p*
Annual incidence of clinical mastitis		0.017
≤1% (3/29, 10.3%)	reference	-
>1% (11/37, 29.7%)	3.667 (0.916–14.685)	0.07
Use of detergent for parlour cleaning after the milking sessions		0.034
Yes (13/65, 20.0%)	reference	-
No (1/1, 100.0%)	11.667 (0.450–302.720)	0.14

^1^ odds ratios calculated against the lowest prevalence associations of variables.

**Table 7:** Results of multivariable analysis for assessment of the isolation of multi-resistant staphylococci from teatcups of milking parlours in 66 goat herds in Greece.

Variable	Odds Ratio ^1^ (95% Confidence Intervals)	*p*
Use of detergent for parlour cleaning afterthe milking sessions		0.035
Yes (10/65, 15.4%)	reference	-
No (1/1, 100%)	15.857 (0.604–416.359)	0.10

^1^ odds ratio calculated against the lowest prevalence associations of variables.

## Data Availability

Most data presented in this study are in the Supplementary Materials. The remaining data are available on request from the corresponding author. The data are not publicly available as they form part of the Ph.D. thesis of the first author, which has not yet been examined, approved, and uploaded in the official depository of Ph.D. theses from Greek Universities.

## Supplementary Materials

The following are available online at https://www.mdpi.com/article/10.3390/antibiotics12091428/s1, Table S1: Results (frequencies) of univariable analysis of variables evaluated for association with the outcome ‘isolation of staphylococci from teatcups’ in the milking parlours of 66 goat herds in Greece; Table S2: Frequency of susceptibility/resistance to individual antibiotics of staphylococcal isolates recovered from the teatcups in the milking parlour of 66 goat herds in Greece; Table S3: Results (frequencies) of univariable analysis of variables evaluated for association with the outcome ‘isolation of oxacillin-resistant staphylococcal isolates from teatcups of a milking parlour’ in 66 goat herds in Greece; Table S4: Results (frequencies) of univariable analysis of variables evaluated for association with the outcome ‘isolation of resistant staphylococcal isolates from teatcups of a milking parlour’ in 66 goat herds in Greece; Table S5: Results (frequencies) of univariable analysis of variables evaluated for association with the outcome ‘isolation of multi-resistant staphylococcal isolates from teatcups of a milking parlour’ in 66 goat herds in Greece; Table S6: Variables evaluated for potential association with recovery of staphylococci from teatcups of milking parlours of 66 goat herds in Greece; Table S7: Details of multivariable models employed for the evaluation of predictors for the recovery of staphylococcal isolates from teatcups of milking parlours in 66 goat herds in Greece.
