# Supplementary material for: Recovery of Staphylococci from Teatcups in Milking Parlours in Goat Herds in Greece: Prevalence, Identification, Biofilm Formation, Patterns of Antibiotic Susceptibility, Predictors for Isolation
Source: Antibiotics (Basel). 2023 Sep 10;12(9):1428. doi: 10.3390/antibiotics12091428 (PMC10525802; doi:10.3390/antibiotics12091428)
Supplement: Supplementary file 1 [file antibiotics-12-01428-s001.zip › antibiotics-2558121-supplementary.pdf]

Recovery of Staphylococci from Teatcups in Milking Parlours in Goat Herds in Greece: Prevalence, Identification, Biofilm Formation, Patterns of Antibiotic Susceptibility, Predictors for Isolation

Charalambia K. Michael, Daphne T. Lianou, Katerina Tsilipounidaki, Dimitris A. Gougoulis, Themistoklis Giannoulis, Natalia G. C. Vasileiou, Vasia S. Mavrogianni, Efthymia Petinaki and George C. Fthenakis

Table S1. Results (frequencies) of univariable analysis of variables evaluated for association with the outcome 'isolation of staphylococci from teatcups' in the milking parlours of 66 goat herds in Greece.

Season of the year when sampling was performed

Herds from which staphylococci were not isolated (n = 31)				Herds from which staphylococci were isolated (n = 35)				
Spring	Summer	Autumn	Winter	Spring	Summer	Autumn	Winter	p
12	11	4	4	7	15	3	10	0.22

Month into the lactation period at sampling

Herds from which staphylococci were not isolated (n = 31)				Herds from which staphylococci were isolated (n = 35)				
0 – 1st	2nd – 5th	6th – 9th	After 9th	0 – 1st	2nd – 5th	6th – 9th	After 9th	p
2	13	12	4	3	14	15	3	0.92

Management system applied in farms

Herds from which staphylococci were not isolated (n = 31)				Herds from which staphylococci were isolated (n = 35)				
Intensive	Semi-intensive	Semi-extensive	Extensive	Intensive	Semi-intensive	Semi-extensive	Extensive	p
5	11	14	1	3	11	20	1	0.72

No. of does in the herd

Herds from which staphylococci were not isolated (n = 31)				Herds from which staphylococci were isolated (n = 35)				
≤ 165 does	166 - 330 does	331 - 500 does	> 500 does	≤ 165 does	166 - 330 does	331 - 500 does	> 500 does	p
20	6	2	3	17	9	6	3	0.46

Age of kid removal from their dams

Herds from which staphylococci were not isolated (n = 31)			Herds from which staphylococci were isolated (n = 35)			
< 45 days	45 – 60 days	> 60 days	< 45 days	45 – 60 days	> 60 days	p
12	11	8	8	15	12	0.37

Average age of culling females

Herds from which staphylococci were not isolated (n = 31)		Herds from which staphylococci were isolated (n = 35)		
≤ 5 years	> 5 years	≤ 5 years	> 5 years	p
11	20	8	27	0.26

Daily number of milking sessions

Herds from which staphylococci were not isolated (n = 31)			Herds from which staphylococci were isolated (n = 35)			
One	Two	Three	One	Two	Three	p
1	29	1	1	30	4	0.45

**Application of post-milking teat disinfection**								
Yes	No	Yes	No					*p*
6	25	5	30					0.58
**Annual milk production per animal**								
≤ 200 L	201 – 400 L	> 400 L	≤ 200 L	201 – 400 L	> 400 L			*p*
13	12	6	18	15	2			0.23
**Annual incidence of clinical mastitis**								
≤ 1%	> 1%	≤ 1%	> 1%					*p*
17	14	12	23					0.09
**Years since initial establishment or most recent renovation of the milking parlour**								
Up to 3 years	4–6 years	Over 6 years	Up to 3 years	4–6 years	Over 6 years			*p*
14	8	9	15	7	13			0.75
**Volume of the parlour**								
Up to 200 m^3	Over 200 m^3	Up to 200 m^3	Over 200 m^3					*p*
21	10	22	13					0.68
**Material of the floor of the milking parlour**								
Concrete	Tiles	Soil	Other	Concrete	Tiles	Soil	Other	*p*
17	5	6	3	18	4	7	6	0.81
**Type of milking parlour**								
Circular	Linear parallel	Linear one-sided	Circular	Linear parallel	Linear one-sided			*p*
1	9	21	3	12	20			0.54
**Type of milking system**								
Built-in	Mobile	Built-in	Mobile					*p*
29	2	30	5					0.30

**Number of animal positions in the parlour**						
Less than 24	24	Over 24	Less than 24	24	Over 24	*p*
12	11	8	11	15	9	0.78
**Number of available milking units per animal position**						
< 1		1	< 1		1	*p*
21		10	21		14	0.51
**Provision of feed in the parlour**						
Yes		No	Yes		No	*p*
27		4	31		4	0.85
**Availability of automated milk quantity measurement**						
Yes		No	Yes		No	*p*
0		31	3		32	0.10
**Availability of milk quality indicators**						
Yes		No	Yes		No	*p*
0		31	0		35	n/a
**System pulsation rate**						
< 140 p. min^{-1}	140 - 150 p. min^{-1}	> 150 p. min^{-1}	< 140 p. min^{-1}	140 - 150 p. min^{-1}	> 150 p. min^{-1}	*p*
9	17	5	10	20	5	0.97
**System pressure**						
< 38 kPa	38 - 42 kPa	> 42 kPa	< 38 kPa	38 - 42 kPa	> 42 kPa	*p*
2	27	2	3	28	4	0.72
**System pulsation rate to pressure ratio**						
< 3.10	3.10–3.79	≥ 3.80	< 3.10	3.10–3.79	≥ 3.80	*p*
7	13	11	8	16	11	0.93

**Type of flow line**						
High	Low	Other	High	Low	Other	*p*
20	8	3	23	8	4	0.95
**Frequency of check-ups of the system by farmer**						
Daily	Less frequently than daily		Daily	Less frequently than daily		*p*
23	8		31	4		0.13
**Annual frequency of check-ups of the system by technicians**						
< 1 check-up	1 - 2 check-ups	> 2 check-ups	< 1 check-up	1 - 2 check-ups	> 2 check-ups	*p*
9	19	3	7	20	8	0.31
**Water cleaning of parlour after the milking sessions**						
Yes	No		Yes	No		*p*
31	0		34	1		0.34
**Temperature of cleaning water**						
≤ 70 °C	> 70 °C		≤ 70 °C	> 70 °C		*p*
19	12		21	14		0.91
**Use of detergent for parlour cleaning after the milking sessions**						
Yes	No		Yes	No		*p*
31	0		34	1		0.34
**Frequency of changing teatcups**						
Annually	Less frequently than annually		Annually	Less frequently than annually		*p*
12	19		17	18		0.42
**Age of the farmer**						
Up to 50 years	Over 50 years		Up to 50 years	Over 50 years		*p*
22	9		25	10		0.97

**Length of previous animal farming experience**				
≤ 5 years	> 5 years	≤ 5 years	> 5 years	*p*
8	23	9	26	0.99
**Presence of working staff in the farm**				
Yes	No	Yes	No	*p*
9	22	17	18	0.10

Table S2. Frequency of susceptibility / resistance to individual antibiotics [1] of staphylococcal isolates recovered from the teatcups in the milking parlour of 66 goat herds in Greece.

	n	AMP [2]	AZI	CXI	CIP	CLA	CLI	ERY	FOS	FUS	GEN	MOX	MUP	OXA	PEN	RIF	STM	TEI	TET	TOB	SXT
S. aureus	6	0	0	0	0	0	0	0	0	0	0	0	0	0	0	0	1	0	0	0	0
S. auricularis	1	0	0	0	0	0	0	0	1	0	0	0	0	0	0	0	0	0	0	0	0
S. capitis	11	1	0	0	0	0	0	1	3	0	0	0	0	0	1	0	0	0	3	0	0
S. caprae	1	0	0	0	0	0	0	0	1	0	0	0	0	0	0	0	0	0	1	0	0
S. cohnii subsp. *urealyticum*	1	1	0	0	0	0	0	0	0	1	0	0	0	0	1	0	0	0	0	0	0
S. epidermidis	1	0	0	0	0	0	0	0	0	0	0	0	0	0	0	0	0	0	0	0	0
S. equorum	10	4	0	0	1	0	4	4	2	1	0	1	0	1	4	0	0	0	1	1	0
S. haemolyticus	7	1	0	0	0	0	1	1	1	0	0	0	0	0	1	0	0	0	1	0	0
S. kloosii	1	0	0	0	0	0	0	0	0	0	0	0	0	0	0	0	0	0	0	0	0
S. lentus	6	2	0	0	2	0	2	1	0	1	0	1	0	2	2	0	0	0	0	0	0
S. pasteuri	4	1	0	0	0	0	0	0	0	0	0	0	0	1	1	0	0	1	0	0	0
S. pettenkoferi	2	0	0	0	0	0	0	0	0	0	0	0	0	0	0	0	0	0	0	0	0
S. saprophyticus	6	2	0	0	0	0	1	1	3	2	0	0	0	0	2	0	0	0	0	0	0
S. sciuri	3	1	0	0	0	0	1	1	1	0	0	0	0	0	1	0	0	0	1	0	0
S. simulans	2	1	0	0	0	0	0	0	0	1	0	0	0	0	1	0	0	0	1	0	0
S. warneri	4	0	0	0	0	0	0	0	0	0	0	0	0	0	0	0	0	0	0	0	0
S. xylosus	1	1	0	0	0	0	1	0	0	0	0	0	0	0	1	0	0	0	1	0	0
Total	67	15	0	0	3	0	10	9	12	6	0	2	0	4	15	0	1	1	9	1	0

[1] as established by use of VITEK 2; [2] AMP: ampicillin, AZI: azithromycin, CXI: cefoxitin, CIP: ciprofloxacin, CLA: clarithromycin, CLI: clindamycin, ERY: erythromycin, FOS: fosfomycin, FUS: fucidic acid, GEN: gentamicin, MOX: moxifloxacin, MUP: mupirocin, OXA: oxaxillin, PEN: penicillin, RIF: rifampicin, STM: streptomycin, TEI: teicoplanin, TET: tetracycline, TOB: tobramycin, SXT: trimethoprim-sulfamethoxazole.

Table S3. Results (frequencies) of univariable analysis of variables evaluated for association with the outcome 'isolation of oxacillin-resistant staphylococcal isolates from teatcups of a milking parlour' in 66 goat herds in Greece.

**Herds from which oxacillin-resistant staphylococci were not isolated (*n* = 62)**				**Herds from which oxacillin-resistant staphylococci were isolated (*n* = 4)**				
**Season of the year when sampling was performed**								
Spring	Summer	Autumn	Winter	Spring	Summer	Autumn	Winter	*p*
18	25	7	12	1	1	0	2	0.50
**Month into the lactation period at sampling**								
0 – 1st	2nd – 5th	6th – 9th	After 9th	0 – 1st	2nd – 5th	6th – 9th	After 9th	*p*
5	24	26	7	0	3	1	0	0.52
**Management system applied in farms**								
Intensive	Semi-intensive	Semi-extensive	Extensive	Intensive	Semi-intensive	Semi-extensive	Extensive	*p*
8	20	32	2	0	2	2	0	0.80
**No. of does in the herd**								
≤ 165 does	166 - 330 does	331 - 500 does	> 500 does	≤ 165 does	166 - 330 does	331 - 500 does	> 500 does	*p*
36	13	8	5	1	2	0	1	0.28
**Age of kid removal from their dams**								
< 45 days	45 – 60 days	> 60 days		< 45 days	45 – 60 days	> 60 days		*p*
19	23	20		1	3	0		0.26
**Average age of culling females**								
≤ 5 years	> 5 years			≤ 5 years	> 5 years			*p*
18	44			1	3			0.86
**Daily number of milking sessions**								
One	Two	Three		One	Two	Three		*p*
2	55	5		0	4	0		0.78

Application of post-milking teat disinfection

Yes	No	Yes	No	*p*
11	51	0	4	0.36

Annual milk production per animal

≤ 200 L	201 – 400 L	> 400 L	≤ 200 L	201 – 400 L	> 400 L	*p*
28	26	8	3	1	0	0.47

Annual incidence of clinical mastitis

≤ 1%	> 1%	≤ 1%	> 1%	*p*
29	33	0	4	0.07

Collaboration with a veterinarian

Yes	No	Yes	No	*p*
50	12	4	0	0.33

Means of calculating live bodyweight for the administration of pharmaceutical products

Weighing	Estimation	Weighing	Weighing	*p*
16	46	1	3	0.97

Routine overdosing of pharmaceuticals

Yes	No	Yes	No	*p*
14	48	2	2	0.21

Annual frequency of systemic disinfections in the farm

0 – 1 occasion	2 - 10 occasions	> 10 occasions	0 – 1 occasion	2 - 10 occasions	> 10 occasions	*p*
11	44	7	1	2	1	0.63

Administration of 'dry-ewe' treatment at the end of the lactation period

Yes	No	Yes	No	*p*
11	51	1	3	0.72

**Years since initial establishment or most recent renovation of the milking parlour**								
Up to 3 years	4–6 years	Over 6 years	Up to 3 years	4–6 years	Over 6 years			*p*
27	15	20	2	0	2			0.51
**Volume of the parlour**								
Up to 200 m³	Over 200 m³	Up to 200 m³	Over 200 m³					*p*
41	21	2	2					0.51
**Material of the floor of the milking parlour**								
Concrete	Tiles	Soil	Other	Concrete	Tiles	Soil	Other	*p*
31	9	13	9	4	0	0	0	0.29
**Type of milking parlour**								
Circular	Linear parallel	Linear one-sided	Circular	Linear parallel	Linear one-sided			*p*
4	19	39	0	2	2			0.67
**Type of milking system**								
Built-in	Mobile	Built-in	Mobile					*p*
55	7	4	0					0.48
**Number of animal positions in the parlour**								
Less than 24	24	Over 24	Less than 24	24	Over 24			*p*
23	23	16	0	3	1			0.24
**Number of available milking units per animal position**								
< 1	1	< 1	1					*p*
39	23	3	1					0.63
**Provision of feed in the parlour**								
Yes	No	Yes	No					*p*
54	8	4	0					0.44

**Availability of automated milk quantity measurement**						
Yes	No		Yes	No		*p*
3	59		0	4		0.65
**Availability of milk quality indicators**						
Yes	No		Yes	No		*p*
0	62		0	4		n/a
**System pulsation rate**						
< 140 p. min^{-1}	140 - 150 p. min^{-1}	> 150 p. min^{-1}	< 140 p. min^{-1}	140 - 150 p. min^{-1}	> 150 p. min^{-1}	*p*
19	34	9	0	3	1	0.41
**System pressure**						
< 38 kP	38 - 42 kPa	> 42 kPa	< 38 kP	38 - 42 kPa	> 42 kPa	*p*
5	51	6	0	4	0	0.65
**System pulsation rate to pressure ratio**						
< 3.10	3.10–3.79	≥ 3.80	< 3.10	3.10–3.79	≥ 3.80	*p*
15	26	21	0	3	1	0.37
**Type of flow line**						
High	Low	Other	High	Low	Other	*p*
40	15	7	3	1	0	0.77
**Frequency of check-ups of the system by farmer**						
Daily	Less frequently than daily		Daily	Less frequently than daily		*p*
50	12		4	0		0.33
**Annual frequency of check-ups of the system by technicians**						
< 1 check-up	1 - 2 check-ups	> 2 check-ups	< 1 check-up	1 - 2 check-ups	> 2 check-ups	*p*
15	37	10	1	2	1	0.89

**Water cleaning of parlour after the milking sessions**				
Yes	No	Yes	No	*p*
61	1	4	0	0.80
**Temperature of cleaning water**				
≤ 70 °C	> 70 °C	≤ 70 °C	> 70 °C	*p*
39	23	1	3	0.13
**Use of detergent for parlour cleaning after the milking sessions**				
Yes	No	Yes	No	*p*
61	1	4	0	0.80
**Frequency of changing teatcups**				
Annually	Less frequently than annually	Annually	Less frequently than annually	*p*
28	34	1	3	0.43
**Age of the farmer**				
Up to 50 years	Over 50 years	Up to 50 years	Over 50 years	*p*
45	17	2	2	0.33
**Length of previous animal farming experience**				
≤ 5 years	> 5 years	≤ 5 years	> 5 years	*p*
16	46	1	3	0.97
**Presence of working staff in the farm**				
Yes	No	Yes	No	*p*
23	39	3	1	0.13

Table S4. Results (frequencies) of univariable analysis of variables evaluated for association with the outcome 'isolation of resistant staphylococcal isolates from teatcups of a milking parlour' in 66 goat herds in Greece.

Season of the year when sampling was performed

Herds from which resistant staphylococci were not isolated (*n* = 52)				Herds from which resistant staphylococci were isolated (*n* = 14)				
Spring	Summer	Autumn	Winter	Spring	Summer	Autumn	Winter	*p*
18	16	6	12	1	10	1	2	0.043

Month into the lactation period at sampling

Herds from which resistant staphylococci were not isolated (*n* = 52)				Herds from which resistant staphylococci were isolated (*n* = 14)				
0 – 1st	2nd – 5th	6th – 9th	After 9th	0 – 1st	2nd – 5th	6th – 9th	After 9th	*p*
5	22	20	5	0	5	7	2	0.56

Management system applied in farms

Herds from which resistant staphylococci were not isolated (*n* = 52)				Herds from which resistant staphylococci were isolated (*n* = 14)				
Intensive	Semi-intensive	Semi-extensive	Extensive	Intensive	Semi-intensive	Semi-extensive	Extensive	*p*
8	17	26	1	0	5	8	1	0.35

No. of does in the herd

Herds from which resistant staphylococci were not isolated (*n* = 52)				Herds from which resistant staphylococci were isolated (*n* = 14)				
≤ 165 does	166 - 330 does	331 - 500 does	> 500 does	≤ 165 does	166 - 330 does	331 - 500 does	> 500 does	*p*
29	11	8	4	8	4	0	2	0.40

Age of kid removal from their dams

Herds from which resistant staphylococci were not isolated (*n* = 52)			Herds from which resistant staphylococci were isolated (*n* = 14)			
< 45 days	45 – 60 days	> 60 days	< 45 days	45 – 60 days	> 60 days	*p*
18	20	14	2	6	6	0.29

Average age of culling females

Herds from which resistant staphylococci were not isolated (*n* = 52)		Herds from which resistant staphylococci were isolated (*n* = 14)		
≤ 5 years	> 5 years	≤ 5 years	> 5 years	*p*
16	36	3	11	0.49

Daily number of milking sessions

Herds from which resistant staphylococci were not isolated (*n* = 52)			Herds from which resistant staphylococci were isolated (*n* = 14)			
One	Two	Three	One	Two	Three	*p*
2	46	4	0	13	1	0.75

**Application of post-milking teat disinfection**						
Yes	No		Yes	No		*p*
11	41		0	14		0.06
**Annual milk production per animal**						
≤ 200 L	201 – 400 L	> 400 L	≤ 200 L	201 – 400 L	> 400 L	*p*
25	20	7	6	7	1	0.67
**Annual incidence of clinical mastitis**						
≤ 1%	> 1%		≤ 1%	> 1%		*p*
26	26		3	11		0.06
**Collaboration with a veterinarian**						
Yes	No		Yes	No		*p*
40	12		14	0		0.047
**Means of calculating live bodyweight for the administration of pharmaceutical products**						
Weighing	Estimation		Weighing	Weighing		*p*
11	41		6	8		0.10
**Routine overdosing of pharmaceuticals**						
Yes	No		Yes	No		*p*
11	41		5	9		0.26
**Annual frequency of systemic disinfections in the farm**						
0 – 1 occasion	2 - 10 occasions	> 10 occasions	0 – 1 occasion	2 - 10 occasions	> 10 occasions	*p*
9	37	6	3	9	2	0.88
**Administration of ‘dry-ewe’ treatment at the end of the lactation period**						
Yes	No		Yes	No		*p*
9	43		3	11		0.72

**Years since initial establishment or most recent renovation of the milking parlour**						
Up to 3 years	4–6 years	Over 6 years	Up to 3 years	4–6 years	Over 6 years	*p*
24	13	15	5	2	7	0.31

**Volume of the parlour**				
Up to 200 m^3	Over 200 m^3	Up to 200 m^3	Over 200 m^3	*p*
35	17	8	6	0.48

**Material of the floor of the milking parlour**								
Concrete	Tiles	Soil	Other	Concrete	Tiles	Soil	Other	*p*
28	6	12	6	7	3	1	3	0.39

**Type of milking parlour**						
Circular	Linear parallel	Linear one-sided	Circular	Linear parallel	Linear one-sided	*p*
3	14	35	1	7	6	0.23

**Type of milking system**				
Built-in	Mobile	Built-in	Mobile	*p*
48	4	11	3	0.14

**Number of animal positions in the parlour**						
Less than 24	24	Over 24	Less than 24	24	Over 24	*p*
18	22	12	5	4	5	0.54

**Number of available milking units per animal position**				
< 1	1	< 1	1	*p*
34	18	8	6	0.57

**Provision of feed in the parlour**				
Yes	No	Yes	No	*p*
45	7	13	1	0.52

**Availability of automated milk quantity measurement**						
Yes	No		Yes	No		*p*
2	50		1	13		0.60
**Availability of milk quality indicators**						
Yes	No		Yes	No		*p*
0	52		0	14		n/a
**System pulsation rate**						
< 140 p. min^{-1}	140 - 150 p. min^{-1}	> 150 p. min^{-1}	< 140 p. min^{-1}	140 - 150 p. min^{-1}	> 150 p. min^{-1}	*p*
15	29	8	4	8	2	0.99
**System pressure**						
< 38 kP	38 - 42 kPa	> 42 kPa	< 38 kP	38 - 42 kPa	> 42 kPa	*p*
5	42	5	0	13	1	0.45
**System pulsation rate to pressure ratio**						
< 3.10	3.10–3.79	≥ 3.80	< 3.10	3.10–3.79	≥ 3.80	*p*
11	23	18	4	6	4	0.82
**Type of flow line**						
High	Low	Other	High	Low	Other	*p*
35	12	5	8	4	2	0.76
**Frequency of check-ups of the system by farmer**						
Daily	Less frequently than daily		Daily	Less frequently than daily		*p*
40	12		14	0		0.046
**Annual frequency of check-ups of the system by technicians**						
< 1 check-up	1 - 2 check-ups	> 2 check-ups	< 1 check-up	1 - 2 check-ups	> 2 check-ups	*p*
12	33	7	4	6	4	0.30

**Water cleaning of parlour after the milking sessions**				
Yes	No	Yes	No	*p*
51	1	14	0	0.60
**Temperature of cleaning water**				
≤ 70 °C	> 70 °C	≤ 70 °C	> 70 °C	*p*
33	19	7	7	0.36
**Use of detergent for parlour cleaning after the milking sessions**				
Yes	No	Yes	No	*p*
52	0	13	1	0.05
**Frequency of changing teatcups**				
Annually	Less frequently than annually	Annually	Less frequently than annually	*p*
24	28	5	9	0.48
**Age of the farmer**				
Up to 50 years	Over 50 years	Up to 50 years	Over 50 years	*p*
39	13	8	6	0.19
**Length of previous animal farming experience**				
≤ 5 years	> 5 years	≤ 5 years	> 5 years	*p*
15	37	2	12	0.27
**Presence of working staff in the farm**				
Yes	No	Yes	No	*p*
20	32	6	8	0.77

Table S5. Results (frequencies) of univariable analysis of variables evaluated for association with the outcome 'isolation of multi-resistant staphylococcal isolates from teatcups of a milking parlour' in 66 goat herds in Greece.

**Herds from which resistant staphylococci were not isolated (*n* = 55)**				**Herds from which resistant staphylococci were isolated (*n* = 11)**				
**Season of the year when sampling was performed**								
Spring	Summer	Autumn	Winter	Spring	Summer	Autumn	Winter	*p*
18	19	6	12	1	7	1	2	0.28
**Application of post-milking teat disinfection**								
Yes		No		Yes		No		*p*
11		44		0		11		0.10
**Annual incidence of clinical mastitis**								
≤ 1%		> 1%		≤ 1%		> 1%		*p*
26		29		3		8		0.22
**Collaboration with a veterinarian**								
Yes		No		Yes		No		*p*
43		12		11		0		0.09
**Means of calculating live bodyweight for the administration of pharmaceutical products**								
Weighing		Estimation		Weighing		Estimation		*p*
12		43		5		6		0.10
**Type of milking system**								
Built-in		Mobile		Built-in		Mobile		*p*
50		5		9		2		0.37
**Frequency of check-ups of the system by farmer**								
Daily (n = 54)		Less frequently than daily (n = 12)		Daily (n = 54)		Less frequently than daily (n = 12)		*p*
43		12		11		0		0.09

**Use of detergent for parlour cleaning after the milking sessions**				
Yes (n = 65)	No (n = 1)	Yes (n = 65)	No (n = 1)	*p*
55	0	10	1	0.025
**Age of the farmer**				
Up to 50 years (n = 47)	Over 50 years (n = 19)	Up to 50 years (n = 47)	Over 50 years (n = 19)	*p*
40	15	7	4	0.54

Table S6. Variables evaluated for potential association with recovery of staphylococci from teatcups of milking parlours of 66 goat herds in Greece.

Parameters related to sampling conditions
Season of the year when sampling was performed (season of the year)
Month into the lactation period at sampling (month)
Parameters related to the management and the health and production in the herds
Management system applied in the herd (description according to EFSA classification)
No. of does in the herd (no.)
Age of kid removal from their dams (days)
Average age of culling females (years)
Daily number of milking sessions (no.)
Application of post-milking teat disinfection (yes / no.)
Annual milk production per animal (L)
Annual incidence of clinical mastitis in the herd (%)
Collaboration with a veterinarian (yes / no)
Means of calculating live bodyweight for the administration of pharmaceutical products (weighing / estimation)
Routine overdosing (compared to dose prescribed) of pharmaceuticals (yes / no)
Annual frequency of systemic disinfections in the farm (no. of occasions)
Administration of 'dry-ewe' treatment at the end of the lactation period (yes / no)
Parameters related to the milking parlour in the herds
Years since initial establishment or most recent renovation of the milking parlour (no.)
Volume of the parlour (m^3)
Material of the floor of the milking parlour (cement / tile / soil / other)
Type of milking parlour (fishbone / circular / linear parallel / linear one-sided / other)
Type of milking system (mobile / non-mobile)
Number of animal positions in the parlour (no.)
Number of available milking units per animal position (no.)
Provision of feed in the parlour (yes / no)
Availability of automated milk quantity measurement
Availability of milk quality indicators
System pulsation rate (p. min^{-1})
System pressure (kPa.)
System pulsation rate to pressure ratio
Type of flow line (low / high / other)
Frequency of check-ups of the system by farmer (description)
Annual frequency of check-ups of the system by technicians (no. of occasions)
Water cleaning of parlour after the milking sessions (yes / no)
Temperature of cleaning water (^oC)
Use of detergent for parlour cleaning after the milking sessions (yes / no)
Frequency of changing teatcups (description)
Parameters related to the socio-demographic characteristics of farmers
Age of the farmer (years)

Length of animal farming experience of the farmer (years)
Presence of working staff in the herd (yes / no)

Table S7. Details of multivariable models employed for the evaluation of predictors for the recovery of staphylococcal isolates from teatcups of milking parlours in 66 goat herds in Greece.

Outcome	Variables offered to the multivariable models (*n*)	Variables required in the final models
Isolation of staphylococci from teatcups	4	(a) Annual incidence of clinical mastitis, (b) Availability of automated milk quantity measurement, (c) Frequency of check-ups of the system by farmer
Isolation of oxacillin-resistant staphylococcal isolates from teatcups of a milking parlour	3	(a) Annual incidence of clinical mastitis, (b) Temperature of cleaning water
Isolation of resistant staphylococcal isolates from teatcups of a milking parlour	9	(a) Application of post-milking teat disinfection, (b) Annual incidence of clinical mastitis, (c) Collaboration with a veterinarian, (d) Frequency of check-ups of the system by farmer, (e) Use of detergent for parlour cleaning after the milking sessions
Isolation of multi-resistant staphylococcal isolates from teatcups of a milking parlour	5	(a) Application of post-milking teat disinfection, (b) Frequency of check-ups of the system by farmer, (c) Use of detergent for parlour cleaning after the milking sessions
